# Supplementary material for: Analysis of PPARγ Signaling Activity in Psoriasis
Source: Int J Mol Sci. 2021 Aug 10;22(16):8603. doi: 10.3390/ijms22168603 (PMC8395241; doi:10.3390/ijms22168603)
Supplement: Supplementary file 1 [file ijms-22-08603-s001.zip › Supplemental materials_Analysis of PPARg signaling activity in psoriasis/Pathway models/Models images and html files/Model of PPARG signaling in psoriasis/13347.html]

RIPK1


# Protein RIPK1

|  |  |
| --- | --- |
| URN | urn:agi-llid:8737 |
| Total Entities | 0 |
| Connectivity | 2755 |
| Name | RIPK1 |
| Description | receptor (TNFRSF)-interacting serine-threonine kinase 1 |
| Notes | This gene encodes a member of the receptor-interacting protein (RIP) family of serine/threonine protein kinases. The encoded protein plays a role in inflammation and cell death in response to tissue damage, pathogen recognition, and as part of developmental regulation. RIPK1/RIPK3 kinase-mediated necrosis is referred to as necroptosis. Genetic disruption of this gene in mice results in death shortly after birth. [provided by RefSeq, Aug 2017] |
| Primary Cell Localization | Cytoplasm |
| Class | Protein kinase |
| ObjectType | Protein |

---

|  |  |
| --- | --- |
| Pathway | mTOR Signaling Hyperfunction |
|  | SIRT2 Signaling in Aging |
|  | TLR4 Signaling in Leukocytes |
|  | Peripheral T-Cell Tolerance |
|  | T-Cell Cytotoxic Mediated Cell Death |
|  | Necrosis |
|  | Eosinophil Survival by Cytokine Signaling |
|  | Apoptosis |
|  | SCF/FBXO7 Complex |
|  | Myocardial Infarction |
|  | Th0-Cell Aberrant Activation in Systemic Lupus Erythematosus |
|  | Podocyte Dysfunction in Diabetic Nephropathy |
|  | Oxidation of Fatty Acid in non-Alcoholic Fatty Liver Disease |
|  | beta-Cell Destruction in Diabetes Mellitus Type 1 |
|  | Hashimoto's Thyroiditis |
|  | Apoptosis of Thyroid Cell in Hashimoto's Thyroiditis |
|  | T-Cell Cytotoxic Response in Ulcerative Colitis |
|  | Hodgkin Lymphoma |
|  | Splenic Marginal Zone Lymphoma |
|  | TNFR/NF-kB Classical Pathway in Multiple Myeloma Plasma Cells |
|  | Multiple Myeloma |
|  | Dendritic Cell Dysfunction in Psoriatic Arthritis |
|  | Osteoclast Activation in Psoriatic Arthritis |
|  | FAS Mediated Apoptosis in Systemic Scleroderma |
|  | Dentin and Enamel Dysruprion in Bloch-Sulzberger Syndrome |
|  | HPV E6 and E7 Induced Disruption of TNFR/NF-kB Signaling in Keratinocytes |
|  | OPTN Effects on NF-kB Signaling in Glaucoma |
|  | OPTN Associated Glaucoma |
|  | Podocyte Dysfunction in Glomerulonephritis |
|  | Apoptosis of Renal Tubule Epithelial Cell in Pyelonephritis |
|  | NFKB Canonical Signaling Activation in Cancer |
|  | STAT3 and NFkB Activate Inflammation-Induced Tumorigenesis |
|  | NFKB Signaling Activation by Blocking of Tumor Suppressors |
|  | Apoptosis Block by Death Receptor Signaling in Cancer |
|  | Apoptosis Evasion in Cancer: Overview |
|  | Effector T-cell Inactivation in Cancer Immune Escape |
|  | TNF-alpha/TNFRSF1A Signaling |
|  | TNF-alpha/TNFRSF1B Signaling |
|  | NF-kB Canonical Signaling |
|  | Model of PPARG signaling in psoriasis |
|  | sars-cov binding |
|  | 1\_Inflammation in keratinocytes in the pathology of acne |
|  | 1\_TNF and IL1B provoke ECM degradation in osteoarthritis |
|  | 1\_Osteoclast-mediated bone resorption in postmenopausal women |
|  | 2\_Chondrocyte death in osteoarthritis |
|  | 2\_Podocyte dysfunction in glomerulonephritis |
|  | 2\_Lipotoxicity in NAFLD |
|  | 6\_p12I Viral protein promotes T-cell activation in HTLV-1 infection |
|  | 5\_Human papilloma virus E6 and E7 proteins promote epithelial cell proliferation |
|  | 1\_Antigen-presenting cells promote islet dysfunction and immune system activation in T1D |

---

|  |  |
| --- | --- |
| Group | binding partnetrs for SARSs |

---

|  |  |
| --- | --- |
| MedScan ID | 8737 |

---

|  |  |
| --- | --- |
| LocusLink ID | 8737 |
|  | 19766 |
|  | 306886 |

---

|  |  |
| --- | --- |
| Alias | Ripk1 |
|  | receptor-interacting protein kinase 1 |
|  | OTTHUMP00000039163 |
|  | receptor (TNFRSF)-interactive serine-threonine kinase I |
|  | receptor-interacting protein 1 |
|  | D330015H01 |
|  | IMD57 |
|  | RIP1 |
|  | Ripk I |
|  | receptor (TNFRSF)-interacting serine-threonine kinase I |
|  | Cell death protein RIP |
|  | RIKEN cDNA D330015H01 gene |
|  | RIP1 kinase |
|  | Serine/threonine protein kinase RIP |
|  | receptor (TNFRSF)-interacting serine-threonine kinase 1 |
|  | receptor (TNFRSF)-interactive serine-threonine kinase 1 |
|  | Rinp |
|  | FLJ39204 |
|  | D330015H01Rik |
|  | RIP |
|  | receptor-interacting serine/threonine-protein kinase 1 |
|  | OTTHUMP00000015955 |
|  | TNFRSF-interacting protein |
|  | TNFRSF interacting protein |
|  | RIP-1 |
|  | serine/threonine-protein kinase RIP |
|  | receptor interacting protein |
|  | Ripk1\_predicted |
|  | Cell death protein RIP |

---

|  |  |
| --- | --- |
| EC Number | 2.7.11.1 |

---

|  |  |
| --- | --- |
| GO ID | 0005524 |
|  | 0004706 |
|  | 0070513 |
|  | 0005123 |
|  | 0004672 |
|  | 0004674 |
|  | 0044877 |
|  | 0031625 |
|  | 0007249 |
|  | 0000165 |
|  | 0002756 |
|  | 0070231 |
|  | 0035666 |
|  | 0007256 |
|  | 0007257 |
|  | 0006919 |
|  | 1990000 |
|  | 0006915 |
|  | 0097190 |
|  | 0044257 |
|  | 0071363 |
|  | 0070301 |
|  | 0071356 |
|  | 0071550 |
|  | 0097191 |
|  | 0070266 |
|  | 0097527 |
|  | 2000134 |
|  | 0043124 |
|  | 0060044 |
|  | 2001237 |
|  | 2001240 |
|  | 1902042 |
|  | 0036289 |
|  | 0043123 |
|  | 0046330 |
|  | 0051092 |
|  | 0043065 |
|  | 2001238 |
|  | 1905206 |
|  | 0032757 |
|  | 0045651 |
|  | 0060545 |
|  | 0010940 |
|  | 1903800 |
|  | 0043068 |
|  | 0001934 |
|  | 2000379 |
|  | 0045944 |
|  | 0032760 |
|  | 1903265 |
|  | 0097300 |
|  | 0046777 |
|  | 0016579 |
|  | 0051291 |
|  | 0051260 |
|  | 0070926 |
|  | 1902041 |
|  | 0010803 |
|  | 0034612 |
|  | 0097343 |
|  | 1901026 |
|  | 0034138 |
|  | 0033209 |
|  | 0016032 |
|  | 0005829 |
|  | 0031264 |
|  | 0010008 |
|  | 0045121 |
|  | 0005739 |
|  | 0043235 |
|  | 0097342 |
|  | 0016301 |
|  | 0000166 |
|  | 0016740 |
|  | 0016310 |
|  | 0043410 |
|  | 0010942 |
|  | 0042327 |
|  | 0012501 |
|  | 2000377 |
|  | 0007165 |
|  | 0005737 |
|  | 0016020 |
|  | 0005886 |
|  | 0032991 |
|  | 0032403 |
|  | 0097296 |
|  | 0010939 |
|  | 0051347 |
|  | 0006468 |
|  | 0043234 |
|  | 0045087 |
|  | 0032481 |
|  | 2001239 |
|  | 0034142 |
|  | 0002224 |
|  | 0016772 |
|  | 0008063 |
|  | 0008633 |
|  | 0006917 |
|  | 0060555 |
|  | 2001243 |
|  | 0019048 |
|  | 0008624 |
|  | 0045768 |
|  | 0010552 |
|  | 0004872 |
|  | 0080010 |
|  | 0005515 |
|  | 0004713 |

---

|  |  |
| --- | --- |
| KEGG ID | hsa:8737 |
|  | mmu:19766 |
|  | rno:306886 |

---

|  |  |
| --- | --- |
| Organism | Homo sapiens {Organism urn:agi-taxid:9606} |
|  | Mus musculus {Organism urn:agi-taxid:10090} |
|  | Rattus norvegicus {Organism urn:agi-taxid:10116} |
|  | Homo sapiens |
|  | Mus musculus |
|  | Rattus norvegicus |

---

|  |  |
| --- | --- |
| Mouse chromosome position | 13 14.01 cM |
|  | 13 13.9 cM |

---

|  |  |
| --- | --- |
| OMIM ID | 603453 |
|  | 618108 |

---

|  |  |
| --- | --- |
| Rat chromosome position | 17p12 |

---

|  |  |
| --- | --- |
| Hugo ID | 10019 |
|  | HGNC:10019 |

---

|  |  |
| --- | --- |
| Human chromosome position | 6p25.2 |

---

|  |  |
| --- | --- |
| Swiss-Prot Accession | A0A024QZU0 |
|  | Q13546 |
|  | Q13546.3 |
|  | Q60855 |
|  | Q60855.1 |
|  | D3ZYL0 |
|  | A0AV89 |
|  | B2RAG1 |
|  | B4E3F9 |
|  | Q13180 |
|  | Q59H33 |
|  | Q3U0J3 |
|  | Q8CD90 |

---

|  |  |
| --- | --- |
| PIR ID | T09479 |
|  | I49299 |

---

|  |  |
| --- | --- |
| GenBank ID | NC\_000006 |
|  | NM\_001317061 |
|  | NP\_001303990 |
|  | NM\_001354932 |
|  | NP\_001341861 |
|  | NM\_003804 |
|  | NP\_003795 |
|  | NM\_001354931 |
|  | NP\_001341860 |
|  | XM\_017011405 |
|  | XP\_016866894 |
|  | NM\_001354934 |
|  | NP\_001341863 |
|  | NM\_001354933 |
|  | NP\_001341862 |
|  | NM\_001354930 |
|  | NP\_001341859 |
|  | XM\_006715237 |
|  | XP\_006715300 |
|  | XM\_017011403 |
|  | XP\_016866892 |
|  | XM\_017011404 |
|  | XP\_016866893 |
|  | NG\_063914 |
|  | AL031963 |
|  | AY682848 |
|  | AAT74626 |
|  | CH471087 |
|  | EAW55115 |
|  | EAW55116 |
|  | EAW55117 |
|  | EAW55118 |
|  | KT584386 |
|  | AB208926 |
|  | BAD92163 |
|  | AJ420482 |
|  | AK096523 |
|  | BAG53315 |
|  | AK304701 |
|  | BAG65471 |
|  | AK314176 |
|  | BAG36858 |
|  | BC126254 |
|  | AAI26255 |
|  | BC126256 |
|  | AAI26257 |
|  | BP228763 |
|  | BX348786 |
|  | CK300756 |
|  | U25994 |
|  | AAC50137 |
|  | U50062 |
|  | AAC32232 |
|  | Q13546 |
|  | NC\_000079 |
|  | XM\_006516607 |
|  | XP\_006516670 |
|  | NM\_009068 |
|  | NP\_033094 |
|  | XM\_006516609 |
|  | XP\_006516672 |
|  | XM\_011244295 |
|  | XP\_011242597 |
|  | NM\_001359997 |
|  | AC161117 |
|  | CH466546 |
|  | EDL40880 |
|  | AK030959 |
|  | BAC27194 |
|  | AK048989 |
|  | AK050301 |
|  | AK086694 |
|  | AK156803 |
|  | BAE33860 |
|  | BC050905 |
|  | AAH50905 |
|  | BC054542 |
|  | AAH54542 |
|  | BC058162 |
|  | AAH58162 |
|  | U25995 |
|  | AAB60487 |
|  | Q60855 |
|  | NC\_005116 |
|  | XM\_017600529 |
|  | XP\_017456018 |
|  | XM\_017600526 |
|  | XP\_017456015 |
|  | XM\_017600528 |
|  | XP\_017456017 |
|  | XM\_017600527 |
|  | XP\_017456016 |
|  | XM\_017600524 |
|  | XP\_017456013 |
|  | XM\_017600525 |
|  | XP\_017456014 |
|  | XM\_017600523 |
|  | XP\_017456012 |
|  | NM\_001107350 |
|  | NP\_001100820 |
|  | AC\_000085 |
|  | AABR07027441 |
|  | AAHX01090952 |
|  | AAHX01090953 |
|  | CH473977 |
|  | EDL98323 |
|  | XM\_005249458 |
|  | XP\_005249515 |
|  | XM\_005249457 |
|  | XP\_005249514 |
|  | XM\_011514969 |
|  | XP\_011513271 |
|  | NC\_018917 |
|  | AMYH02012994 |
|  | HQ258488 |
|  | ADR83242 |
|  | AC\_000035 |
|  | AAHY01105183 |
|  | XM\_006715236 |
|  | XP\_006715299 |
|  | AC\_000138 |
|  | ABBA01048621 |
|  | XM\_006516608 |
|  | XP\_006516671 |
|  | CAD70625 |
|  | NT\_007592 |
|  | NW\_922984 |
|  | NW\_001838972 |
|  | AC\_000049 |
|  | CR618624 |
|  | NT\_039578 |
|  | NW\_001030517 |
|  | NW\_047491 |
|  | NW\_001084723 |
|  | NT\_034880 |
|  | Q59H33 |
|  | XM\_225262 |
|  | XP\_225262 |
|  | XM\_001065998 |
|  | XP\_001065998 |
|  | XM\_006253828 |
|  | XP\_006253890 |
|  | XM\_006253829 |
|  | XP\_006253891 |
|  | AABR06091192 |
|  | AABR06091193 |
|  | AABR06091194 |

---

|  |  |
| --- | --- |
| Swiss-Prot ID | RIPK1\_HUMAN |
|  | RIPK1\_MOUSE |
|  | D3ZYL0\_RAT |

---

|  |  |
| --- | --- |
| Cell Localization | Cytoplasm |
|  | Cell membrane |

---

|  |  |
| --- | --- |
| Ensembl ID | ENSG00000137275 |
|  | ENSP00000259808.3 |
|  | ENST00000259808.9 |
|  | ENSMUSG00000021408 |
|  | ENSMUSP00000129831.1 |
|  | ENSMUST00000167374.8 |
|  | ENSMUSP00000021844.8 |
|  | ENSMUST00000021844.14 |
|  | ENSMUSP00000128511.1 |
|  | ENSMUST00000171137.1 |
|  | ENSRNOG00000017787 |
|  | ENSRNOP00000023962.4 |
|  | ENSRNOT00000023962.5 |
|  | ENSP00000369773 |
|  | ENST00000380409 |
|  | ENSP00000259808 |
|  | ENST00000259808 |
|  | ENSMUSP00000129831 |
|  | ENSMUST00000167374 |
|  | ENSMUSP00000021844 |
|  | ENSMUST00000021844 |
|  | ENSRNOP00000023962 |
|  | ENSRNOT00000023962 |

---

|  |  |
| --- | --- |
| MGI ID | MGI:108212 |
|  | 108212 |

---

|  |  |
| --- | --- |
| RGD ID | 1310158 |

---

|  |  |
| --- | --- |
| Unigene ID | Hs.519842 |
|  | Mm.374799 |
|  | Rn.7572 |

---

|  |  |
| --- | --- |
| Homologene ID | 2820 |

---

|  |  |
| --- | --- |
| Shape | Sickle-vertex |

---

|  |  |
| --- | --- |
| IPI ID | IPI00013773 |
|  | IPI00939198 |
|  | IPI00121472 |
|  | IPI00366094 |

---
